# Fixation and staining methods for macroscopical investigation of the brain

**DOI:** 10.3389/fnana.2023.1200196

**Published:** 2023-06-22

**Authors:** Leonardo Nardi, Michael J. Schmeisser, Sven Schumann

**Affiliations:** ^1^Institute of Anatomy, University Medical Center of the Johannes Gutenberg University, Mainz, Germany; ^2^Focus Program Translational Neurosciences, University Medical Center of the Johannes Gutenberg University, Mainz, Germany

**Keywords:** brain fixation, brain staining, Klingler method, blue berlin method, formalin, neurophobia

## Abstract

The proper preservation of human brain tissue is an indispensable requirement for *post-mortem* investigations. Neuroanatomical teaching, neuropathological examination, neurosurgical training, basic and clinical neuroscientific research are some of the possible downstream applications of brain specimens and, although much apart from one another, proper tissue fixation and preservation is a common denominator to all of them. In this review, the most relevant procedures to fixate brain tissue are described. *In situ* and immersion fixation approaches have been so far the most widespread ways to deliver the fixatives inside the skull. Although most of them rely on the use of formalin, alternative fixative solutions containing lower amounts of this compound mixed with other preservative agents, have been attempted. The combination of fixation and freezing paved the way for fiber dissection, particularly relevant for the neurosurgical practice and clinical neuroscience. Moreover, special techniques have been developed in neuropathology to tackle extraordinary problems, such as the examination of highly infective specimens, as in the case of the Creutzfeldt-Jakob encephalopathy, or fetal brains. Fixation is a fundamental prerequisite for further staining of brain specimens. Although several staining techniques have been developed for the microscopical investigation of the central nervous system, numerous approaches are also available for staining macroscopic brain specimens. They are mostly relevant for neuroanatomical and neuropathological teaching and can be divided in white and gray matter staining techniques. Altogether, brain fixation and staining techniques are rooted in the origins of neuroscience and continue to arouse interest in both preclinical and clinical neuroscientists also nowadays.

## Introduction

Fixation of the brain is a challenging yet needful procedure. While fresh unfixed brains might still be used for specific *post-mortem* investigations (Waldvogel et al., 2006), fixation is unavoidable for neuroanatomical teaching, neuropathological macro- and microscopical enquiries, neurosurgical training and neuroscientific research. In the context of neuroanatomical teaching, implementing neuroanatomical classes with human brain dissection leads to dramatic improvement of short- and long-term retention of knowledge (Rae et al., 2016). Considering that basic neuroscience and neuroanatomy are often perceived as overly complex by the students, leading to the so-called “neurophobia” (Jozefowicz, 1994; Moreno-Zambrano et al., 2021), the availability of high-quality specimens might be key to the improvement of the neuroanatomical teaching. Similar considerations can also be made for macroscopic brain staining methods. The discovery and further development of brain staining techniques has characterized the history of neuroscience from its dawning, enabling the morphological study of the central and peripheral nervous systems. Although the latest advances are more oriented toward the deciphering of neuronal connections at microscopic, circuit and ultrastructural levels (Endo et al., 2021; Luo, 2021), macroscopic staining of brain slices still finds a useful application in neuroanatomical teaching. The development of fiber-dissecting techniques has helped generations of neurosurgeons to develop their skills prior to operation. In recent years, the interest for this approach has risen again (Costa et al., 2018; Altieri et al., 2019; Grzegorczyk et al., 2019; Dziedzic et al., 2023). The combination of classical, neuroanatomical, fiber-dissection techniques and more advanced neuroradiological approaches such as diffusion tensor imaging and fiber tractography, have broadened the understanding of several white-matter connections (Holl et al., 2011; Akakin et al., 2014; Sarubbo et al., 2015; Meola et al., 2016; Burks et al., 2017; Goryainov et al., 2017; Pascalau et al., 2018; Flores-Justa et al., 2019; Shah et al., 2019; Dziedzic et al., 2021a,^2022^; Rauch et al., 2021), even leading to the discovery of novel structures (Shinohara et al., 2020). Throughout the last 150 years, a plethora of fixation and staining methods has been described. In the first part of the review, a historical perspective of the different methods developed will be given, focusing in particular on the approaches used to effectively deliver the fixatives intrathecally (summarized in Table 1). In the second part, we will turn our attention to the most important methods devised to stain the brain for further macroscopical analysis.

**TABLE 1:** Overview of the fixation techniques.

References	Main technical characteristics	Commentaries
**Fixation methods based on formalin**		
Parker and Floyd, 1896	• 60% ethanol (85%) • 40% formalin (2%)	• Protocol optimized to reduce swelling of the brain
Sainton and Kattwinkel, 1898	• Undiluted formalin	• First description of *in situ* fixation through orbita or nose cavities
Lagerlöf and Torgersruud, 1934	• 10% formalin • 90% physiological solution (1.75%)	• Protocol optimized to reduce swelling of the brain
Klingler, 1935	• Immersion fixation in 5% formalin • Freezing at −10 to −15°C	• Milestone method for the fiber-dissection approach • Lengthy procedure (takes up to 2–3 months)
Fahr, 1938	• Undiluted formalin	• *In situ* fixation through a hole drilled close to the ear
Hasenjäger and Spatz, 1938	• Physiological solution (exsanguination step) • Undiluted formalin	• First description of fixation through the internal carotid artery
Ostertag, 1949	• 20% formalin and Carlsbad salt solution (*in situ* fixation) • 10% formalin and 7% Carlsbad salt solution (post-fixation)	• *In situ* fixation through the nose cavities
Böttger, 1951	• Physiological solution • Undiluted formalin	• First description of fixation through the common carotid artery
Khadziselimowitch, 1963	• 10% formalin • 5% formalin • Sugar solution	• Protocol optimized to reduce swelling of the brain
Bradbury and Hoshino, 1978	• 50% Champion Clot Disperser solution in water (pre-injection fluid) • 50% ethylene glycol, 20% methanol, 10% phenol, 20% formalin (embalming fluid)	• *In situ* formalin post-fixation makes brain more suitable for dissection
Anderson, 2006	• Formalin-based method using: • Dodge Metaflow^®^ (pre-injection fluid) • Dodge Metasyn^®^ (embalming fluid)	• Example of light-embalming technique
Latini et al., 2015	• Intracarotid fixation with 12% formalin • Freezing at −15 to −20°C	• Optimization of Klingler’s technique
Insausti et al., 2023	• Physiological solution in 0.1 M phosphate buffer, pH 7.4 + 50000 U heparin (exsanguination step) • 4% paraformaldehyde solution in 0.1 M phosphate buffer (fixation and post-fixation)	• The method permits a wide range of downstream applications
**Fixation methods using reduced formalin or alternative chemical compounds**		
Thiel, 2002	• 40% water • 45% ethanol • 15% formalin	• *In situ* formalin post-fixation through a peristaltic pump makes brain more suitable for dissection
Messmer et al., 2010	• ESCO EPIC conditioner and methanol • Freezing at −18°C	• Exsanguination step preceding the fixing-freezing approach • *In situ* formalin post-fixation makes brain more suitable for dissection
Anichkov et al., 2011	• 10 ± 3% aqueous PHMGH	• Original color and volume retained, elastic consistency
Al-Hayani et al., 2011	• Dry Shellac • Water • Ethanol	• Brains show brownish discoloration
Hammer et al., 2012	• Ethanol-glycerine-thymol fixation method	• *In situ* or immersion formalin post-fixation makes brains more suitable for dissection
**Fixation methods based on formalin**		
Miyake et al., 2020	Comparison of the following brain fixation approaches: 1. Formalin 2. Thiel method 3. Thiel method + intraventricular formalin injection	The third alternative proved more effective for brain dissection
Crosado et al., 2020	• Ethanol-water-glycerine-phenoxyethanol fixation method	• *In situ* formalin post-fixation makes brains more suitable for dissection
**Fixation methods developed to address outstanding problems**		
Brown et al., 1990	• 48 h in formalin • 1 h in formic acid • 48 h in formalin	• Formic acid proved superior to phenol in disinfection and tissue preservation against prion
Nicholls, 1988	• Heidenhain-Susa fixative	• Protocol for fetal brain fixation • The fixative is highly toxic due to the presence of mercury
Bass et al., 1993	• Zamboni solution	• Protocol for fetal brain fixation
Cimmino et al., 2002	• 30% anhydrous acetic acid • 30% formalin (40%) • 40% distilled water	• Protocol for fetal brain fixation • Protocol for fetal brain fixation
Barrett et al., 2004	• 10% formalin	• Microwave application for fast fixation of fetal brains • Internal parts not thoroughly fixed
Sharma and Grieve, 2006	• 20% formalin	• Protocol for rapid fixation

The most relevant technical points are reported. For more details, such as the precise approach and duration of the intrathecal delivery of the fixatives, refer to the main text.

## Fixation methods

In one of the first approaches described by Sainton and Kattwinkel (1898), a trocar, connected to an 80 cm long plastic tube, is inserted either through the orbita or through the nose. A total of 80–100 mL formalin are then injected, leading to an *in situ* fixation of brain and spinal cord. Similarly, 15 mL of 20% formalin and Carlsbad salt solution can be injected through nose and ethmoidal bone, resulting in a hardening of the skull base. The brain can then be extracted and put in a 10% formalin and 7% Carlsbad salt solution for 2–3 weeks (Ostertag, 1949). An alternative way to access the brain is by drilling a hole close to the ear. A cannula for lumbar puncture can then be inserted to the inferior horn of the lateral ventricles. At this point, 60–70 mL of formalin solution can be slowly injected (Fahr, 1938).

An *in situ* fixation of the brain can also be achieved by injecting the fixative solution in one of the arterial blood vessels, which reach the brain. According to Hasenjäger and Spatz (1938), the skull should be removed right after death and the dura mater folded till the superior sagittal sinus. The anterior part of the cerebral falx and of the optical nerve are then severed. The frontal lobe can now be lifted, and the internal carotid artery can be reached inside the skull. A curved cannula can then be inserted through a slit, and warmed physiological solution can be injected at low pressure. The blood will consequently run off from the opened sinus. At this point, formalin solution can be injected. The same procedure can be then repeated with the contralateral internal carotid artery (Hasenjäger and Spatz, 1938). Arteries reaching the brain can also be prepared more proximally. According to Böttger (1951), both common carotid arteries can be bound 2–3 cm below the bifurcation. A plastic catheter is then inserted and held in place with a Kocher-clip. A total of 500–700 mL first of physiological solution, afterward of 20% formalin, are injected. After 30 min the brain can be removed and post-fixed (Böttger, 1951). In order to avoid swelling of brains put in formalin, a solution made up of 6 parts of alcohol 85% and 4 parts of 2% formalin can be used. The final amount of liquid should be 5 times higher than the volume of the brain (Parker and Floyd, 1896). Almost identical results can be achieved using neutral formalin and 1.75% physiological solution in a ratio of 1 to 9 (Lagerlöf and Torgersruud, 1934). Dipping the brain in a sugary solution also enables to maintain the original volume. The brain can be first fixed for 24 h in 10% formalin followed by 6 weeks in 5% formalin. In the end, the brain can be stored in a concentrated sugary solution (Khadziselimowitch, 1963). In the context of a fresh tissue laboratory, the development of a light embalming technique has helped preserving bodies for a longer time, keeping suitable for dissection. The femoral artery and vein are first prepared, and the artery is cannulated. A total of 475 mL of a pre-injection fluid, consisting of Dodge Metaflow^®^ diluted 1:1 with cold water, is injected in the femoral artery. The fluid is allowed to remain in the body for 15 min before the injection fluid is administered. This consists of 475 mL of Dodge Metasyn^®^ diluted 1:16 with cool water. The total volume of the embalming fluid is hence 8.475 L, which is enough for a body between 68 and 82 kg of weight. A total of 25–50 mL of Dodge Icterine Regular^®^, an arterial dye, can be dissolved in 8 L embalming solution. The bodies prepared in this way can then be stored at 4°C prior to use. For the brain, no particular changes of the protocol are requested (Anderson, 2006). Very recently, a new protocol, which has the advantage of permitting several downstream applications such as macroscopical investigation, magnetic resonance imaging, classical histology, immunohistochemistry, and electron-microscopy, has been proposed. Briefly, 4 L of physiological solution in 0.1 M phosphate buffer, at pH 7.4, containing 50000 U of heparin, are injected through the common carotid artery in order to drain off blood rests through the sectioned jugular vein. A total of 8 L of 4% paraformaldehyde in 0.1 M phosphate buffer are then injected over 1 h (the first 4 L in 20 min, the second 4 L in 40 min). The brain is left for 24–48 h *in situ* and only then it is extracted and post-fixed in 3 L 4% paraformaldehyde at 4°C. Brains post-fixed up to 5 years can still be profitably processed (Insausti et al., 2023).

In 1935 Klingler published a capital article, in which he described a new method suitable for the dissection of white fiber tracts. For a historical perspective of Klingler’s work, its importance for the development of neurosurgery and the broad application in neuroanatomical research, we recommend the following reviews (Agrawal et al., 2011; Dziedzic et al., 2021b). Briefly, brain is fixed by immersion in 5% formalin for 2–3 months at least. A freezing step then follows. The brains are kept for 8 to 10 days at −10 to −15°C. Afterward, the brains are thawed in water at room temperature. The freezing procedure leads to a dilatation of about 10% of the fixative. Since the solution does not penetrate the myelinated fibers but only remains between them, the fibers are separated from each other (Zemmoura et al., 2016). The loosening up of the fibers enables eventually their preparation with very smooth, sharp tweezers. The brains can be temporarily stored in 2% formalin solution. More recently, a new method was developed to minimize potential limitations of the Klingler technique, such as the long duration of immersion fixation, deriving possible structural anomalies and the lack of a satisfactory vascular architecture. The brains are first fixed with an intra-carotid injection of 2 L of 12% formalin and extracted 48 h after the perfusion. They are then stored in 10% formalin for 24 h. Upon removal of the leptomeninges, the brains are frozen at −15 to −20°C for 6–10 days and then slowly defrosted for 12 h. The brains might be placed in 5% formalin between dissection sessions (Latini et al., 2015). Even if technically challenging to perform, the Klingler method for fiber dissection has also proven itself as effective in increasing the understanding of three-dimensional brain structures in the context of neuroanatomy teaching (Silva and Andrade, 2016).

Other authors also proved the effectiveness of combining freezing and chemical fixation in preserving human corpses and brains. At Duke University, a fixing-freezing approach, preceded by an exsanguination step, has been used. Exsanguination was achieved by inserting a drain tube in the internal jugular vein exerting suction at 80 mmHg. The additional use of an anticoagulant solution injected through a catheter in the carotid artery enhanced the removal of clotted blood. A mixture of ESCO EPIC conditioner and methanol at a ratio of 1:2 was later injected in the arterial circulation and eventually corpses were stored at −18°C until use. Brain fixation was optimized by injecting 60 mL of 10% formalin through a 16-mm hole drilled in the frontal region (Messmer et al., 2010).

In recent years, there has been a growing research interest toward alternative fixatives solutions, in the attempt to reach a reduced formalin content, securing, at the same time, high quality of tissue preservation. As we will see, for an optimal preservation of brain tissue, an additional step, consisting in injecting formalin in the skull, is still necessary.

The approach described by Thiel offers superlative tissue quality except for central nervous system. In order to obtain optimal results, a special approach should be undertaken after the application of the solutions described (Thiel, 1992). The whole body should be lied face up and the head should dangle from the preparation table. Two cannulas are then inserted through the nose and the ethmoid bone until they reach the anterior horn of the lateral ventricle. The corpse can be now turned face down and lumbar puncture between L5 and S1 can be performed. The nose cannulas are connected to a peristaltic pump, which pumps in the conservation solution, made up of 10% formalin and 40% isopropyl alcohol. The lumbar cannula is then connected to the tank containing the conservation solution. The circulation is hence so closed and the corpse can be perfused for 24 h (Thiel, 2002). In a recent study, brains subjected to formalin fixation, Thiel’s embalming method and Thiel’s embalming method with additional intra-ventricular formalin injection were compared in terms of brain surface stiffness and elasticity. The latter approach showed the best results regarding elasticity and safety, making it suitable for head and brain surgical training (Miyake et al., 2020).

Alternatively, polyhexamethyleneguanidine hydrochloride (PHMGH) was assessed in comparison with formalin as fixative. Brains and organs were extracted and immersion-fixed in a solution consisting of 10 ± 3% aqueous PHMGH for 1 month. Control specimens were fixed with 10% formalin. Organs fixed with PHMGH retained the original color and volume, showing an elastic consistency. The different haptic characteristics may be due to the different chemical mechanism of actions of the two compounds (Anichkov et al., 2011).

Shellac, a natural polymer derived from insects and used in food and pharmaceutical industry, was also tested as possible fixative. Shortly, 80 kg of dry resin are mixed to a solution made up of 120 L ethanol and 80 L water. After preparation of the skull and the breast for an optimal diffusion of the solution, the corpses are put in the above-mentioned solution inside a pressurized tank. The brains treated in this way showed a slightly brownish discoloration (Al-Hayani et al., 2011).

Bradbury and Hoshino (1978) describe a successful approach for long-lasting preservation of cadavers. Both the femoral and the common carotid arteries are cannulated and injected with a blood clot dispersing solution (one bottle of Champion Clot Disperser mixed 1:1 with tap water) for 10–20 min. Afterward, the embalming fluid is infused. This consists of 5 gallons of ethylene glycol, 2 gallons of methanol, 1 gallon of liquefied phenol and 2 parts of undiluted formalin. At this point, both the jugular and the femoral veins are slit open, so that the embalming fluid can drain out. The authors suggest additional treatment for an optimal fixation and preservation of the brains. After drilling one hole at the vertex of the body, 50–100 cm^3^ of undiluted formalin is injected on both hemispheres. Alternatively, 50 cm^3^ of undiluted formalin can be injected bilaterally through the superior orbital fissure (Bradbury and Hoshino, 1978). Hammer et al. (2012) described an ethanol-glycerine based fixation with minimal formaldehyde content, accompanied by thymol conservation. The central nervous system can either be fixed exclusively with ethanol-glycerin or be further exposed to formaldehyde. In the first case, brains appear softened and shrunk. In the second case, after the ethanol-glycerine fixation step, formaldehyde can be either delivered *in situ* (50–100 mL injected in the subdural space after trepanation) or by immersion after removal of the brain. When formaldehyde is applied, brains result indurated, making them more suitable for dissection (Hammer et al., 2012). Phenoxyethanol fixation might represent one of the most promising alternatives to formalin for fixation. Initially, it was successfully used for post-fixation and long-term preservation of formalin-fixed bodies (Frolich et al., 1984; Wineski and English, 1989). More recently, a protocol for the whole-body fixation based on phenoxyethanol was described. Approximately 20 L of the fixation solution, which consists of a mixture of ethanol, water, glycerin and phenoxyethanol, is injected over 4 h through the femoral artery. To obtain optimal brain fixation, 100 mL of the above-mentioned solution containing 20% formalin, need to be injected. Bilateral holes should be drilled 10–15 mm lateral to the median sagittal line, and, after injection, they should be sealed either with wax or with a resin (Crosado et al., 2020).

## Fixation methods developed to address outstanding problems

In order to overcome challenging conditions (e.g., potential prion infection, structural frailty of fetal brains), neuropathologists have developed various approaches to fix brains. In case of suspect prion disease such as Creutzfeldt-Jakob encephalopathy, dipping the brain specimen in formic acid for 1 h, after 48 h immersion in formalin, proved effective in inactivating the prions (Brown et al., 1990). A similar approach may be also used in anatomical institutes in the case of brains derived from donors affected by a not better characterized form of dementia. Following several cases of organ retention in several pathology institutes in Great Britain, a new, faster, protocol for brain fixation (so called rapid fixation) was developed, with the aim to reconcile both solid neuropathological investigation and feelings of the bereaved. The rapid fixation protocol consisted in the application of 20% formalin through both the blood vessels at the skull base and the floor of the third ventricle. The brains were later dipped for 1–4 days in 20% formalin. The previous approach followed by the authors consisted in suspending the brain in 10% formalin for 3–4 weeks, without preceding injection of the fixative in the blood vessels. The section and staining quality of the tissues was compared and no statistically significant difference between the two approaches was found (Sharma and Grieve, 2006).

When dealing with fetal brains, special carefulness is required not to damage frail structures. Although in the German anatomical institutes pediatric body donation is not contemplated, the technical aspects described in the following part of the review might be adapted to the fixation of adult brains. Nicholls injects in the anterior and posterior fontanelle the mercury-containing Heidenhain-Susa fixative. On the following day, the skull is removed, and the brain is put for 3 days in the Heidenhain-Susa fixative. The brain can then be finally washed and used for further investigation (Nicholls, 1988). Bass et al. (1993) first apply a lumbar needle and then inject 120 mL of Zamboni solution in the lateral ventricle. As soon as the solution flows through the lumbar needle, the procedure is repeated in the contralateral lateral ventricle. If the lumbar puncture is not practicable, the needle can be placed in the cisterna magna. The brain can be removed up to 5 days after the fixation and should be then placed in Zamboni solution (Bass et al., 1993). Cimmino et al. (2002) use a solution made up of 3 parts of anhydrous acetic acid, 3 parts of 40% formaldehyde and 4 parts of distilled water. The fixative should be injected through the anterior, posterior, and mastoid fontanelles. After placing the fetus for 20 h in formalin, it is possible to extract the brain for macro- and microscopic examinations (Cimmino et al., 2002). In the attempt to perform a neuropathological examination in short times, so that the funeral must not be overly delayed, a heat accelerated fixation protocol was established for pediatric brains. After removal, the brain is placed in a 5 L basket, submerged with 10% formalin and covered with cotton wool. The fixation takes place in a MicroMed T/T Mega (Milestone, Sorisole, Italy) microwave in two steps. In the first one, 600 W power are applied for 20 min to raise the temperature of the formalin to 50°C. In the second one, 300 W power for 6 h maintain the temperature of the fixative at 50°C. In the end, the brain is left cooling for 12 h, is washed in running water and finally can be dissected. The internal parts of the brain are not thoroughly fixed though (Barrett et al., 2004). For a broader overview about the available methods for the fixation of fetal and perinatal brains, we recommend (van Dijk et al., 2021).

In the context of brain banking, a great number of perfusion fixation approaches and fixatives have been described. For an overview we recommend (McFadden et al., 2019; McKenzie et al., 2022).

## Staining techniques for macroscopical investigation of the brain

Upon successful fixation of brain specimens, macroscopic sections might be further stained for downstream applications, the most important being neuroanatomical teaching and neuropathological investigation. In neuroanatomical teaching institutions, brain slices are stained in order to highlight structures, which will be then explained to the students. Stained specimens can then be plastinated for long-term conservation and reutilization (Suriyaprapadilok and Withyachumnarnkul, 1997; Baeres and Moller, 2001; Asadi et al., 2013). In neuropathology, brain macroscopic staining approaches are paradigmatically employed to investigate the extensiveness of the white and gray matter damage in demyelinating diseases (Moore et al., 2000; Geurts and Barkhof, 2008; Reynolds et al., 2011; Moller et al., 2019). In the next part of the review, the most important approaches aimed at staining gray and white matter of the brain will be recapitulated (summarized in Table 2).

**TABLE 2:** Overview of the staining techniques for brain gray and white matters.

References	Main technical characteristics	Commentaries
**Gray matter staining methods based on the reaction with iron, other metals or non-metallic substances**		
Sincke, 1926	• 1% iron chloride solution • 1% potassium ferrocyanide solution	• Relatively long incubation time (4–12 h). • Slight toxicity of potassium ferrocyanide
Obersteiner, 1924	• 2.5% solution of potassium dichromate • 90% ethanol	• Potassium dichromate is highly toxic
Kondratjew, 1926	• Vanadium anhydrite in 5% solution of glycerophosphoric acid • 1–1.5% toluidine red	• Time consuming (several days) • Staining must be performed at body temperature
Mainland, 1928	• 1% iron chloride solution • 1% potassium ferrocyanide solution • 1% HCl solution	• Adjustment of Sincke’s protocol (faster incubation times)
Blair et al., 1932	• Sodium sulfide and cobalt nitrate • Sodium sulfide and lead nitrate • Antimony potassium tartrate and hydrogen sulfide • Lead nitrate and potassium iodide • Soluble starch solution and iodine solution in potassium iodide	• The study can be conceived as a proof of concept for the several combinations of metals tested. The brain slices are not permanently stained.
**Mulligan’s method of gray matter staining methods and variations**		
Mulligan, 1931	• Mulligan’s phenol solution: 4% phenol, 0.5% copper sulfate and 0.125% hydrochloric acid • 2% tannic acid • 2% iron alum	• Incubation in the Mulligan’s solution should take place at 60°C
Green, 1933	• Same as Mulligan, 1931	• Incubation in the Mulligan’s solution takes place at room temperature instead of 60°C
Le Masurier, 1935	• Mulligan’s phenol solution • 1% iron chloride solution • 1% potassium ferrocyanide solution	• Combination of Mulligan’s protocol (incubation in hot phenol) and Sincke’s protocol (use of potassium ferrocyanide for the staining)
Brown, 1939	• Mulligan’s phenol solution • 1% lead nitrate • 1% silver nitrate • Some drops of concentrated nitric acid • 5–10% ammonium sulfide	• Optimization of Mulligan’s and Blair et al. (1932) methods for teaching purposes
Tompsett, 1955	• Mulligan’s phenol solution • 10 s in ice water • 2% iron chloride solution • 1% ferrocyanide solution • Storage solution: 75 mL distilled water, 25 mL glycerin, 10 mL formalin and 0,2 g citric acid	• The brain slices should be protected from light for at least 1 month
Roberts and Hanaway, 1969	• Mulligan’s phenol solution • 2% potassium ferrocyanide solution	• Phenol concentration of the Mulligan solution was increased from 4 to 5% • Tannic acid and iron alum steps were removed
Gregg, 1975	From Mulligan’s method the following were changed: • Reduction of tannic acid from 2 to 0.4% • Substitution of iron alum with 0.08% ferric ammonium sulfate	• Optimization of Mulligan’s protocol for plastic embedding
**Gray matter staining methods based on the reaction with iron, other metals or non-metallic substances**		
Alston, 1981	• Mulligan’s phenol solution • 1% polyclens in xylene solution • 2% sodium hydroxide • 2% potassium ferrocyanide	• Protocol optimized for batch staining
Barnett et al., 1980	• Same as in Tompsett, 1955 • 25% acetic acid solution in water or in 10% formalin	• Improved sectioning through gelatin embedding • Protocol optimized for plastic embedding • Post-fixation of the brain in 25% anhydrous acetic acid significantly inhibits dye reduction
**Gray matter staining methods based on CPTS**		
Braak, 1978	• Performic acid: 0.1 L H_2_O_2_ (30%) + 0.9 L formic acid (98–100%) • Staining solution: 0.1 g astra blue copper phthalocyanine and 0.01 L HCl (37%) in 1 L distilled water	• Requires several months of fixation in 4% formalin • Stained brain slices show no sign of fading after 3 years
Wu and Kiernan, 2001	• Mulligan’s phenol solution • 1% CPTS solution • 0.5% acetic acid	• No sign of fading up to 4 years
Loftspring et al., 2008	• Comparison between Tompsett’s and CPTS methods, pre-treated or not with phenoxyethanol	• Avoidance of phenol exposure increases the gray matter staining quality.
**White matter staining methods**		
Proescher, 1927	• Oil red o pyridine in 70% ethanol	• Faster than Sudan IV staining
Brody and Wirth, 1957	• 1% Sudan black B in 70% ethanol	• Protocol optimized for plastic embedding of the brain slices
Dziabis, 1958	• Staining solution: 1 g Luxol fast blue, 1 L 95% ethanol, 5 mL 10% acetic acid • 10 mL lithium carbonate diluted in 1 L 70% ethanol	• Staining needs to be performed at 45–50°C
Hewitt, 1959	• 0.5 g of Sudan III or Sudan IV in a mixture of equal volumes of 70% ethanol and acetone	• The whole procedure lasts up to 1 year

The most relevant technical points are reported. For more details, such as the fixation applied, washing steps, incubation times and storage, refer to the main text.

## Staining of the gray matter

In an early study about the iron content in the extrapyramidal motor system, Spatz noted that placing unfixed brain slices in a solution of ammonium hydrosulfide led to gray staining of several regions, acknowledging the presence of iron in the central nervous system (Spatz, 1922). A great number of staining techniques reported here rely on the reaction of certain chemicals with cerebral iron, which leads to chromatic differentiation of gray and white matter (Kramer, 1936).

The first report about selective staining of the gray matter dates to 1926. Slices obtained from brains fixed in formalin are immersed in 1% iron chloride solution for 4–12 h depending on the thickness, washed in distilled water and in 1% potassium ferrocyanide solution. The slices are then again washed in water and stored in formalin. The gray matter of brains stained with this approach appears deep blue, whereas the white matter pale blue (Sincke, 1926). Shortly after, a development of Sincke’s protocol was proposed, with the finality of making the stained sections more permanent. The brain slices fixed in formalin were cut and washed in current water. Then, they were immersed for 25 s in a 1% iron chloride solution and once again washed in current water. In the next step, they were put for 5 s in 1% potassium ferrocyanide and once more washed. Finally, they were incubated in 1% HCl and stored either in 4% formalin or 70% ethanol. With this technique, the gray matter appears light blue and the white matter remains unstained (Mainland, 1928). In order to increase the contrast between gray and white matter, Mulligan developed a new approach, which has proved to be one of the most successful for the staining of the gray matter. Care was taken regarding removing as much blood as possible from the brain, since it can spoil the contrast between gray and white matter. The brains were then fixed in Kaiserling solution, cut with a sharp knife, and washed again with running water overnight. The brain slices were additionally fixed in 10% formalin, washed first in running water for 12 h and then in distilled water for 1 h. Before staining the brain slices, a treatment must be undertaken in order to produce a protective layer over the white matter. The slices should be placed for 2 min in a mixture made up of 4% phenol, 0.5% copper sulfate and 0.125% hydrochloric acid (Mulligan’s phenol solution) at a temperature of 60°C. The brain slices should then be washed in cold water for 1 min. After the formation of the protective film, the brain slices can be stained. They are immersed in 2% tannic acid for 1 min, washed in running water for 5 min and finally in 2% iron alum in the form of violet crystals dissolved in distilled water, until the gray matter turns black. At this point, the slices should be washed for 24 h in running water and then be further processed for long term storage (Mulligan, 1931). The protocol described has been widely used and modified in the following years. We will report in the next section an overview of the most relevant evolutions of the Mulligan’s method.

Blair et al. (1932) have demonstrated that Mulligan’s results can be obtained (even if not permanently) also with other color reactions that, instead of involving iron salts, rely either on other metals or non-metallic substances. The brains, fixed and hardened in formalin, were first immersed in the first-named reactants, washed, and then placed in the second-named reactant. The chemical product of the reaction between first- and second-named reactants accounts for the color. The reactant combinations are here listed:

• Sodium sulfide and cobalt nitrate; the precipitation of cobalt sulfide stains the gray matter gray black.
• Sodium sulfide and lead nitrate; the precipitation of lead sulfide tinges the gray matter dark brown.
• Antimony potassium tartrate and hydrogen sulfide; the precipitation of antimony sulfide dyes the gray matter orange yellow.
• Lead nitrate and potassium iodide; the precipitation of lead iodide turns the gray matter canary yellow.
• Finally, soluble starch solution and iodine solution in potassium iodide; the white matter becomes pale yellow, whereas the gray matter dark purple (Blair et al., 1932).

Brown proposes to first fix brains by infusing 10% formalin through the circle of Willis and then post-fix in formalin for 10–14 days. After washing with water, slices can be cut. They are then fixed again for 8 h in 10% formalin and re-washed in running water for 12 to 24 h. At this point, brain slices can be placed for 1–2 min in 5% phenol at 60–70°C, for 5–10 min in warm water (40–50°C) and then in cold water for a few minutes. The sections are now ready to be stained for 1 min in a solution made up of equal parts of 1% lead nitrate and 1% silver nitrate with some drops of concentrated nitric acid. After washing in running water for 10–15 min, the slices can be immersed for about 15 s in 5–10% ammonium sulfide solution. After a final washing step, the slices can be either embedded or stored in 10% formalin (Brown, 1939).

**FIGURE 1:**
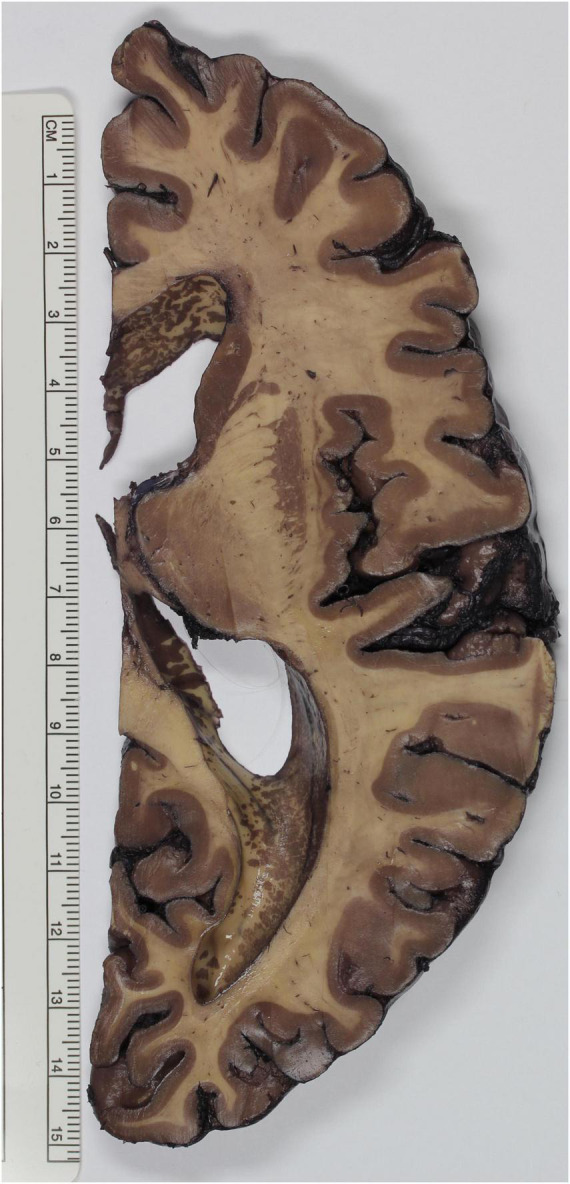
Brain specimen stained according to the method described by Gregg. The gray matter of the telencephalic cortex, basal ganglia and thalamus appears deep brown, whereas white matter tracts, such as the internal capsule and the semioval center, emerge as faintly stained.

Green modified Mulligan’s method performing the step with phenol at room temperature instead of 60°C, obtaining good results for neuroanatomical teaching (Green, 1933). In 1935 Le Masurier futher improved Green’s protocol by treating the brain slices first with hot phenol and using then Sincke’s solution for staining (Le Masurier, 1935). Tompsett (1955) also modified Mulligan’s protocol, in the following ways: the slices were put for 5 min in the Mulligan’s phenol solution, for 10 s in ice water and for 40–60 s in a 2% iron chloride solution. This reagent induces a light brown coloring of the gray matter. The given time should not be exceeded, otherwise the white matter could get stained. The slices should then be washed for 1 min in running water. At this point, the slices were immersed in 1% ferrocyanide potassium solution, which turns the iron chloride in Prussian blue. After leaving over night the slices in water, a strong water jet should be applied in order to remove a membrane-like deposit, result of the chemical reactions. The slices should be protected for at least 1 month from light, in a solution made up of 75 mL distilled water, 25 mL glycerin, 10 mL formalin and 0,2 g citric acid. Finally, they can either be stored in this solution for longer periods or be embedded (Tompsett, 1955). Another evolution of Mulligan’s approach can be found in Roberts and Hanaway’s protocol. They removed the iron-alum and tannic acid steps and implemented potassium ferrocyanide. Brain fixation should be optimized by perfusing with 40% formalin after removal and successive storage in 10% formalin for 2–4 weeks. After cutting (preferably 4 mm thickness), slices should be washed for 12 h in running water. The concentration of phenol of Mulligan’s phenol solution is increased to 5%. Brain slices are immersed in this solution at 60°C for 6 min, washed again in running water for 5 min and finally treated with 2% potassium ferrocyanide for 30–60 s until the gray matter turns red brown. After washing again for 5 min in water, the stained slices can be stored in 10% formalin (Roberts and Hanaway, 1969). A further adaptation of this protocol was also developed, in order to make batch staining possible. Brains completely fixed in formalin are washed 2 to 3 days in running water and embedded in 20% aqueous gelatin, which is left hardening overnight. At this point, the hemispheres can be cut, and the slices stored between sheets of water-resistant photographic paper (Kodak), placed in 1% phenoxyethanol solution, a bactericidal and fungicidal compound that does not affect the further reactions of the protocol. After washing in water for 25–30 min, the slices of one hemisphere can be loaded on a steel mesh tray, which is stained for immersion in a 20 L tank. First, the slices are placed for 20 min in a solution made up of 750 mL of 80% phenol, 75 g of copper sulfate, 15 mL HCl and 15 L water. Contrarily to Mulligan, this solution is kept at room temperature. The slices are then immersed for 20 s with agitation in the second solution, which consists of 15 L xylene and 150 mL polyclens. This treatment eliminates mottling of the white matter. The slices should then be moved immediately to the third solution, containing 300 g sodium hydroxide in 15 L water, again in agitation, for 10 s. Treatment with sodium hydroxide increases the hydrophobic properties of the white matter and enhances the penetration of the dye in the tissue. As last, brain slices are moved for 1 to 2 min to a container with 15 L water and 300 g potassium ferrocyanide. This compound reacts with the cupric sulfate forming cupric ferrocyanide, which stains the cortex red brown. In order to stop the reaction, the brain slices are washed in running water for 5 min and stored for long term in 2% formalin (Alston, 1981). Gregg modified Mulligan’s approach to overcome difficulties encountered upon plastic embedding. The concentration of tannic acid is reduced from 2 to 0.4% and iron alum is substituted by 0.08% ferric ammonium sulfate for 10–15 s (Gregg, 1975; Figure 1). Moreover, exposure to light or to elevated temperatures reduce the blue color to green, leading also to a decrease of contrast between gray and white matter. Another protocol was proposed to sort out this issue by Barnett et al. (1980). Brains fixed in formalin were first embedded in gelatin so that precisely oriented serial sections could be obtained. The slices were washed in running water at 65–80°C to remove gelatin and then stained as described by Tompsett (1955). After rinsing in water, a post-fixation step, consisting in 24–48 h immersion in 25% anhydrous acetic acid or 25% anhydrous acetic acid in 10% formalin, was added. This last step was proven effective in prolonging the vivacity of the blue color (Barnett et al., 1980). Other authors also used either Mulligan’s or Le Masurier’s approach, modifying them slightly (Barnard et al., 1949; Foglmann, 1959).

To circumvent some drawbacks related to Mulligan’s protocol such as the lengthy procedure and the fading of the staining over several years, new approaches using copper phthalocyanine or its derivate copper phthalocyanine tetrasulfonic acid tetrasodium salt (CPTS) were developed. According to Braak (1978), brains should be kept in 4% formalin for several months before being processed. Brain slices should be washed in water and placed in performic acid for 1 h. After washing in running water overnight, the slices should be placed for 12–24 h in a solution of copper phthalocyanine dye astra blue (^®^Merck) made up of 0.1 g astra blue die in 1 L water, to which 10 mL HCl (37%) should be added. After washing the brains for 1 h, slices can be embedded. Brain slices treated with this approach showed no sign of fading after 3 years (Braak, 1978). A variation of this protocol was shortly after produced. Instead of astra blue, 0.05% solution of copper phthalocyanine alcian blue, which stains glycosaminoglycans in the gray matter, was used as a dye (Heller and Stoddard, 1986). Wu and Kiernan used brains fixed by immersion in 4% formalin for several months, which were cut into 1 cm thick slices. Initially, the brain slices were immersed in Mulligan’s phenol solution for 5 min at 60°C, then for 1 min in 10°C cold water. In a following step, the brain slices were put for 10–15 min in 20–25°C warm 1% CPTS solution. In the end, the brain slices were washed at least 15 min in running water and finally stored in 0.5% acetic acid solution. Brain slices treated with this protocol show a turquoise-blue staining of the gray matter and no fading for up to 48 months (Wu and Kiernan, 2001). In a following study this protocol was tested in combination with a phenoxyethanol storage and phenol treatment. After phenol staining CPTS could effectively stain the gray matter in brains not stored in phenoxyethanol, whereas significant white matter staining was detected in brains exposed to phenoxyethanol. Both brains exposed and not exposed to phenoxyethanol yielded a good gray matter staining quality and little white matter staining, if the phenol exposure, typical of the Mulligan’s approach, was avoided (Loftspring et al., 2008).

Although Mulligan’s method and variations of it have taken a predominant role, other protocols based on different approaches are also available. A compound for the staining of central and peripheric nervous system was for example described by Kondratjew (1926). The procedure to produce it takes several days and it should be injected at body temperature. The compound consists of a mixture of vanadium anhydrite in a 5% solution of glycerophosphoric acid. The mixture is left for 2 days until it turns yellow (phosphorous acid). Iron powder should be slowly added to the solution, which is then left for 2–3 days. Until here, all the procedures should be performed at warm temperatures. Finally, twice or three times as much water is mixed, and everything is filtered. To the filtrate, 1–1.5 g toluylene red is added. The solution is left for 2–3 days warming before use (Kondratjew, 1926). Obersteiner pickled brains fixed in formalin in a 2.5% solution of potassium dichromate and then cut them. After a thorough wash with water, the slices were placed in 90% ethanol. Adding HCl can improve the staining differences between gray and white matter. The characteristic green staining is preserved by storing the brain slices in diluted glycerin (Obersteiner, 1924).

## Staining of the white matter

Some proceedings for the whole brain staining with liposoluble dyes such as Sudan are also available. In general, these dyes penetrate in regions with high content of myelin, leading to a coloration of the white matter.

**FIGURE 2:**
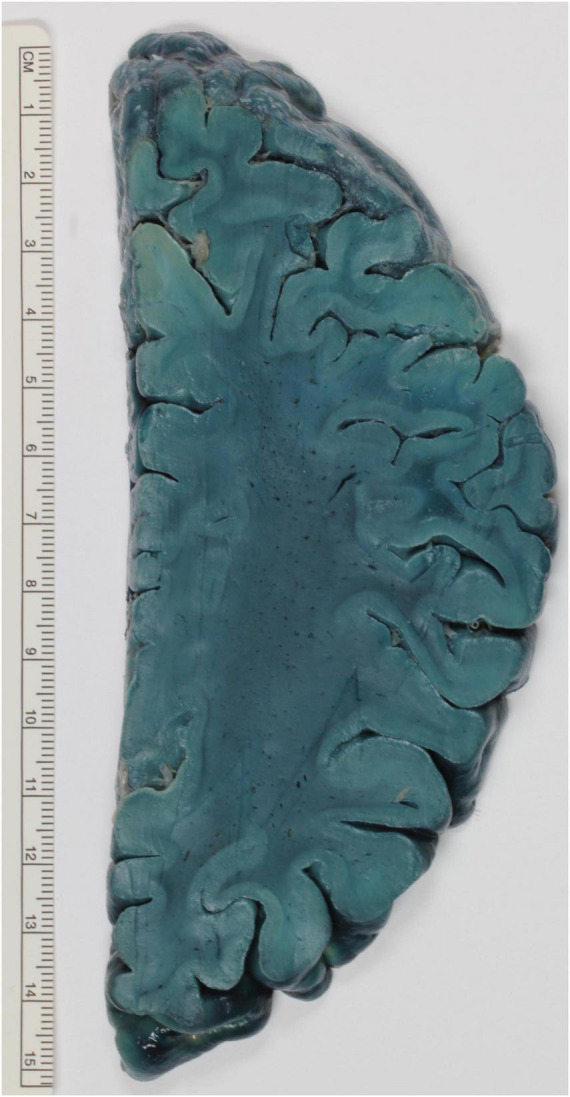
Exemplary brain slice stained according to the method described by Brody and Wirth. The white matter is strongly stained and stands out against the paler cortex.

In Proescher (1927) praised the fast-staining properties of Oil Red O Pyridin, which can color in 30 min brain samples to deep orange red color. In comparison, Sudan III stainings require several hours for the same results. The same dye (2 mg dissolved in 128 cm^3^ benzene) was used for brain slices. The stain penetrates 1 to 2 mm in the tissue. Although fixation with formalin improves the stain quality, such fixation is not necessary (Waldman and Michaels, 1954). To overcome the reduced retainment of Oil Red O Pyridin in plastic embedded brain slices, Brody and Wirth developed the following protocol. After fixation of the brain with 10% formalin, the brain is cut and washed in water and then put in the solution for 1 h. This consists of 1% Sudan black B dissolved in 95% ethanol, eventually diluted to 70%. The brain should be then destained as desired in 70% ethanol and then be immersed for 1 h in 50% ethanol. At this point, the section can either be stored indefinitely in 10% formalin or embedded (Brody and Wirth, 1957). An example of this protocol can be seen in Figure 2. A similar procedure was developed independently by Hewitt. Whole brains are fixed either in 70% alcohol or 10% formaldehyde. The staining solution is made up of either Sudan III or Sudan IV dissolved in a mixture of equal volumes of 70% ethanol and acetone. A total of 0.5 g of Sudan III or IV to every 100 mL should be calculated. The volume of the staining solution should be ten times the volume of the brain, which should remain immersed for at least 6 months. For an equal amount of time, the staining should be differentiated in 70% ethanol. Initially, the ethanol should be changed weekly or when it turns to deep red color. The differentiation is then terminated when the ethanol only slowly changes to orange red. Finally, the brain can be stored in 70% ethanol or 10% formalin, in particular if it has to be dissected (Hewitt, 1959).

Thanks to the method based on Luxol fast blue developed by Dziabis (1958), the gray matter appears green or very pale gray and the white matter bright blue. The brains are fixed in 10% formalin and cut in 2–5 mm thick slices. They are then re-fixed for 1–2 weeks, washed for at least 6 h in tap water and dehydrated by placing twice for 1 h in 95% ethanol. The staining takes place for 16–18 h in a solution made up of 1 g Luxol fast blue diluted in 1 L 95% ethanol and 5 mL 10% acetic acid at 45–50°C. Excessive dye can be removed by washing in 95% ethanol and water. The slices are then immersed in 10 mL saturated lithium carbonate solution in 1 L distilled water and 70% ethanol. After washing in water, the slices can be stored in 10% formalin (Dziabis, 1958).

## Conclusion

In this review the most relevant fixation and staining techniques for the macroscopic study of brains were discussed. Although many reports date back to the first part of the past century, interest in the field stays sparkling. A comparative study on the long-term preservation of brain slices stained with several of the methods described in the text, was recently performed in the Anatomical Institute in Magdeburg (Fischer, 2019). In neurosurgery, interest in Klinger’s dissection method is strong, because of the possibilities derived from practicing before real operations (Dziedzic et al., 2021b). The importance of proper brain fixation is also of paramount relevance for designing brain banks, in which brain material is further investigated through several microscopic and biochemical approaches (McFadden et al., 2019). Lastly, the availability of well-preserved and well-dissected brain specimens can be accounted as a decisive factor in enhancing the effective studying of neuroanatomy and in contrasting the spread phenomenon of “neurophobia” (Pakpoor et al., 2014; Tarolli and Jozefowicz, 2018). Investigation of effective fixation and conservation methods for the brain is hence also nowadays unavoidable for the development of neuroscience.

## Author contributions

LN produced the first draft of the manuscript. MS and SS critically revised and edited the manuscript. All authors approved the manuscript for publication.
